# In Vivo Imaging of *oskar* mRNA Transport Reveals the Mechanism of Posterior Localization

**DOI:** 10.1016/j.cell.2008.06.053

**Published:** 2008-09-05

**Authors:** Vitaly L. Zimyanin, Katsiaryna Belaya, Jacques Pecreaux, Michael J. Gilchrist, Alejandra Clark, Ilan Davis, Daniel St Johnston

**Affiliations:** 1 The Gurdon Institute and the Department of Genetics, University of Cambridge, Tennis Court Road, CB2 1QN Cambridge, UK; 2 Wellcome Trust Centre for Cell Biology, University of Edinburgh, Mayfield Road, Edinburgh EH9 3JR, UK

**Keywords:** CELLBIO, DEVBIO, RNA

## Abstract

*oskar* mRNA localization to the posterior of the *Drosophila* oocyte defines where the abdomen and germ cells form in the embryo. Although this localization requires microtubules and the plus end-directed motor, kinesin, its mechanism is controversial and has been proposed to involve active transport to the posterior, diffusion and trapping, or exclusion from the anterior and lateral cortex. By following *oskar* mRNA particles in living oocytes, we show that the mRNA is actively transported along microtubules in all directions, with a slight bias toward the posterior. This bias is sufficient to localize the mRNA and is reversed in *mago*, *barentsz*, and *Tropomyosin II* mutants, which mislocalize the mRNA anteriorly. Since almost all transport is mediated by kinesin, *oskar* mRNA localizes by a biased random walk along a weakly polarized cytoskeleton. We also show that each component of the *oskar* mRNA complex plays a distinct role in particle formation and transport.

## Introduction

mRNA localization is a common mechanism for targeting proteins to specific regions of a cell and plays an important role in axis formation in many organisms, where localized mRNAs function as cytoplasmic determinants. This has been extensively studied in *Drosophila*, where both main body axes are defined by the localization of *bicoid*, *oskar* (*osk)*, and *gurken* mRNAs to distinct regions of the oocyte (Bashirullah et al., 1998; St Johnston, 2005). *osk* mRNA moves to the posterior of the oocyte during stages 8–10 of oogenesis and is translated as soon as it is localized to the posterior pole, where Oskar protein nucleates the polar granules, which contain the abdominal and germline determinants (Ephrussi et al., 1991; Kim-Ha et al., 1991, 1995; Markussen et al., 1995).

The *Drosophila* egg chamber consists of a syncytium of 15 nurse cells that are connected by ring canals to the oocyte. *osk* mRNAs are transcribed in the nurse cells and must first be transported into the oocyte. This localization depends on the activity of BicD and Egalitarian, which are thought to couple the mRNA to dynein, which moves the mRNAs toward the minus ends of microtubules in the oocyte (Bullock and Ish-Horowicz, 2001; Clark et al., 2007). The localization of *osk* mRNA within the oocyte is also microtubule dependent and is disrupted by treatments with microtubule depolymerizing drugs (Clark et al., 1994).

Microtubule stainings or live imaging of microtubule-associated proteins reveal that the oocyte contains an anterior-posterior gradient of microtubules during the stages when *osk* mRNA is localized, in which most microtubules are nucleated from the anterior and lateral cortex. In addition, a fusion between the plus end-directed motor, kinesin, and β-galactosidase accumulates at the oocyte posterior, suggesting that plus ends are enriched at this pole (Clark et al., 1994). However, it has been argued that the Kinβgal fusion protein localizes to the posterior by hitchhiking since microtubules cannot be detected at the oocyte posterior (Cha et al., 2002). Thus, the organization of the microtubules in the oocyte is still controversial, and it is unclear how the microtubule network directs *osk* mRNA to the posterior.

Several mutants disrupt *osk* mRNA localization within the oocyte without affecting the cytoskeleton and are good candidates for mutants in essential *trans*-acting factors. These include three factors (HRP48, the exon junction complex [EJC], and Staufen) that colocalize with *osk* mRNA both before and after its localization and whose posterior localization is *osk* mRNA dependent, strongly suggesting that they are essential components of the *osk* mRNA localization complex. Null mutants in the hnRNPA/B protein, HRP48, are cell lethal, but three point mutations lead to a uniform distribution of *osk* mRNA in the oocyte (Huynh et al., 2004). Mutants in the EJC components, Mago nashi, Y14, eIF4AIII, and Barentsz, lead to the mislocalization of the mRNA to the anterior of the oocyte (Hachet and Ephrussi, 2001; Mohr et al., 2001; Newmark and Boswell, 1994; Palacios et al., 2004). The EJC is recruited to mRNAs during splicing and marks where exons have been removed. Consistent with this, the first intron of *osk* is necessary for its posterior localization (Hachet and Ephrussi, 2004). Mutants in the dsRNA-binding protein, Staufen (Stau), cause a similar accumulation of *osk* mRNA at the anterior of the oocyte to EJC mutants, but a small amount of RNA is always localized to the posterior at stage 9 (Ephrussi et al., 1991; Kim-Ha et al., 1991; St Johnston et al., 1991; van Eeden et al., 2001). This localization is only transient, however, because Stau is also required for the translational activation of *osk* mRNA and its anchoring at the posterior pole (Micklem et al., 2000). While the EJC and HRP48 disappear from the oocyte posterior at stage 10 of oogenesis, Stau associates with *osk* mRNA throughout oogenesis (St Johnston et al., 1991; Jenny et al., 2006).

As well as these RNA-binding proteins, Tropomyosin II (TmII) and the Kinesin heavy chain (Khc) are also required for *osk* mRNA localization. A P-element allele of *TmII* leads to the mislocalization of *osk* mRNA to the anterior (Erdelyi et al., 1995). Tropomyosin stabilizes F-actin and modulates its interaction with other actin-binding proteins, but there is no other evidence for a direct role of actin in *osk* mRNA localization, and Tropomyosin does not colocalize with *osk* mRNA. Null alleles of the *Khc* cause a different phenotype, in which *osk* RNA localizes around the anterior and lateral cortex (Brendza et al., 2000; Cha et al., 2002). Kinesin is enriched at the oocyte posterior at stage 9, although this localization is not *osk* mRNA dependent, unlike the RNA-binding proteins mentioned above (Palacios and St Johnston, 2002).

The role of the Khc suggests a simple model for *osk* mRNA localization, in which this plus end-directed motor is linked to the mRNA by HRP48, the EJC, and Stau and transports the mRNA along microtubules to the posterior pole. It is not known, however, if kinesin associates with the *osk* mRNA localization complex directly, and the motor is also required for the cytoplasmic flows in the oocyte cytoplasm and for the posterior localization of dynein (Brendza et al., 2002; Januschke et al., 2002; Palacios and St Johnston, 2002). Thus, the requirement for kinesin in *osk* mRNA localization may be indirect.

Glotzer et al. have proposed an alternative model for *osk* mRNA localization, in which the RNA diffuses throughout the oocyte cytoplasm and is selectively captured at the posterior by a prelocalized anchor (Glotzer et al., 1997). This model is based on the observation that injected *osk* mRNA accumulates at the posterior of late-stage oocytes without showing any directed movement toward the posterior pole. Instead, the RNA is washed around the oocyte by the cytoplasmic flows at these stages. Both the strong cytoplasmic flows in late oocytes and the weaker “seething” movements at stage 9 depend on kinesin (Serbus et al., 2005). The motor might therefore play an indirect role in *osk* mRNA localization by generating flows that facilitate its diffusion, thereby ensuring its efficient delivery to a posterior anchor.

Cha et al. have proposed a third model in which kinesin transports *osk* mRNA away from all regions of the cortex except the posterior (Cha et al., 2002). This cortical exclusion model is based on the observation that the mRNA accumulates in the center of the oocyte at stage 8 but is found all round the cortex in *Khc* germline clones. According to the model, *osk* mRNA localization is driven by the lack of microtubules emanating from the posterior cortex, thereby preventing kinesin from removing the RNA from this region. Both the central accumulation of *osk* mRNA at stage 8 and its posterior localization at stage 9 are delayed in *Khc* hypomorphs that reduce the speed of the motor without affecting its other properties (Serbus et al., 2005). This has led to the proposal that kinesin is required for both steps in *osk* mRNA localization: it first transports the mRNA to the center of the oocyte and then facilitates its targeting to the posterior, perhaps by directed transport along a specific subset of microtubules or by catalyzing random movements that are somehow biased toward the posterior.

One way to distinguish between the models for *osk* mRNA localization is to observe its movement in living oocytes. The most common approach for visualizing mRNA localization has been injection of fluorescently labeled transcripts (Cha et al., 2001; MacDougall et al., 2003; Weil et al., 2006; Wilkie and Davis, 2001). This technique cannot be used for *osk* mRNA, however, because splicing of the first intron is required for its localization. An alternative approach is to fuse GFP to an RNA-binding protein that associates with the mRNA of interest, and ZBP-1-GFP and Exu-GFP have been used in this way to track *β-actin* and *bcd* mRNA movements (Oleynikov and Singer, 2003; Theurkauf and Hazelrigg, 1998). One caveat with this method is that most RNA-binding proteins bind to more than one mRNA, making it important to demonstrate that the tagged RNA-binding protein only associates with the appropriate transcript.

An elegant way to solve this problem is to target GFP to the mRNA by inserting binding sites for the MS2 phage coat protein into the mRNA and coexpressing an MS2-GFP fusion protein that also includes a nuclear localization signal (Bertrand et al., 1998). Since MS2 does not bind any endogenous RNAs in *Drosophila*, the fusion protein is targeted to the nucleus unless it binds to the MS2-binding sites in the transcript of interest and is exported with the RNA into the cytoplasm. This method has been used successfully to study *Ash1* mRNA localization in yeast (Beach et al., 1999; Bertrand et al., 1998), *CaMKIIα3* and *β-actin* mRNA localization in cultured mammalian cells (Fusco et al., 2003; Rook et al., 2000), and *nanos* and *bicoid* mRNA in *Drosophila* oocytes (Forrest and Gavis, 2003; Weil et al., 2006).

Here we report the use of two labeling strategies to image the dynamics of *osk* mRNP particles in living oocytes. Our analysis of their movements rules out all of the proposed mechanisms for *osk* mRNA localization and leads us to propose a revised model for how the mRNA is transported to the posterior of the oocyte.

## Results

### Visualization of *osk* mRNPs in Living Oocytes

**Figure 1:**
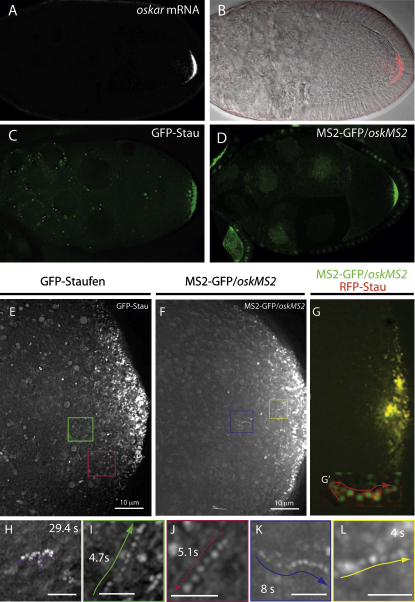
*osk* mRNA Particles Undergo Fast, Directed Movements in All Directions in the Oocyte Cytoplasm (A and B) *osk* mRNA localization to the posterior of a wild-type stage 9 oocyte. (B) An overlay of a pseudo differential interference contrast image and the in situ hybridization signal (red channel) from (A). (C) GFP-Stau localization in a live stage 9 oocyte. (D) The localization of *osk* mRNA labeled with MS2-GFP in a live stage 9 egg chamber. MS2-GFP contains a nuclear localization signal and therefore localizes to the nurse cell nuclei when not associated with *osk* mRNA. (E and F) Overlays of 25 frames from timelapse movies of wild-type stage 9 oocytes expressing GFP-Stau (E) or *oskMS2*/MS2-GFP (F) to show the movements of *osk* mRNA particles. Examples of individual tracks are highlighted by colored rectangles and shown in (I)–(L). (G) *oskMS2/*MS2-GFP and RFP-Stau colocalize at the posterior pole of the oocyte and in individual moving particles. (G′) shows an overlay of 25 frames from a movie in which the red and green channels were imaged alternately. See also Movie S5. (H) An example of a particle moving passively with the cytoplasmic flows and undergoing Brownian motion. (I–L) Closeups of the fast, directed particle tracks, highlighted by the colored rectangles in (G) and (H). GFP-Stau (I and J); *oskMS2*/MS2-GFP (K and L). Scale bars are 2 μm.

We have previously tried to visualize *osk* mRNA by following the movement of a GFP-tagged version of Stau that colocalizes with the mRNA throughout oogenesis and fully rescues the *osk* mRNA localization defects of *stau* null mutants (Figures 1A–1C) (Palacios and St Johnston, 2002). Although we observed large GFP-Stau granules in the oocyte cytoplasm by confocal microscopy, the velocity of these granules never exceeded the speed of cytoplasmic flows (0.1 μm/s), and the granules always moved in the same direction as other organelles in the surrounding cytoplasm. Furthermore, these large granules only form when the protein is overexpressed and cannot be detected by antibody staining of endogenous Stau. This suggested that the large granules are aggregates of overexpressed GFP-Stau, and that the particles containing *osk* mRNA are too faint or too fast to visualize using standard imaging systems.

To overcome this limitation, we imaged oocytes expressing GFP-Stau at high magnification with much faster frame rates (2–4 frames/s) using a sensitive wide-field deconvolution microscope (Figure 1E). This revealed the existence of a population of very small and faint Stau particles throughout the oocyte cytoplasm, which often underwent fast, directed movements at speeds of up to 1 μm/s, consistent with active transport (Figures 1I and 1J; Movie S1 available online). Furthermore, GFP-Stau particles often followed each other along the same track, suggesting that they were moving along the same microtubule or actin filament (Movie S2). Surprisingly, these movements occurred in all directions. In contrast to these fast movements, many particles moved only slowly along irregular paths (>0.1 μm/s), suggesting that they were being carried by the slow cytoplasmic flows and undergoing Brownian motion (Figure 1H and data not shown).

The lack of a clear bias in the direction of GFP-Stau particle movements was unexpected given the models for *osk* mRNA localization and raised the question of whether all of the particles contain *osk* mRNA. Stau associates with other transcripts at other stages of development, and it is therefore possible that some of the particles in the stage 9 oocyte represent Stau complexed with another mRNA, or free Stau aggregates (St Johnston, 2005). We therefore used the MS2 system to label *osk* mRNA directly by generating an *osk* genomic rescuing construct in which 10 MS2-binding sites were inserted immediately after the *osk* stop codon. When this transgene is crossed to flies expressing MS2-nls-GFP, the MS2-GFP-labeled *oskMS2* mRNA accumulates at the posterior of the oocyte in an identical pattern to endogenous *osk* mRNA (Figure 1D). *oskMS2* rescues the early oogenesis defect of an *osk* mRNA null mutant and still localizes normally to the posterior of the oocyte at stage 9 in the absence of endogenous *osk*, confirming that the introduction of MS2-binding sites does not disrupt its localization signal (Figure S1A). Unlike GFP-Stau, *oskMS2* RNA does not form large cytoplasmic aggregates, confirming that the latter are probably an overexpression artifact.

When visualized at high magnification in living oocytes, *oskMS2* mRNA labeled small particles throughout the oocyte cytoplasm, which showed fast movements in all directions similar to those of the small GFP-Stau particles (Movies S3 and S4). Indeed, we could detect even more moving particles of *oskMS2* mRNA, presumably because this labeling system introduces up to 20 GFPs per *osk* mRNA and is therefore more sensitive than the single GFP attached to Stau. Nevertheless, it appears that most if not all small Stau particles contain *osk* mRNA since RFP-Stau particles were also labeled by *oskMS2/*MS2-GFP, and alternate imaging of the red and green channels revealed that the two labels move in concert on the same particle (Figures 1G and 1G′; Movie S5). Thus, GFP-Stau is a reliable marker for *osk* mRNA particles, suggesting that the protein does not associate with other RNAs to form particles at stage 9 of oogenesis.

### Movement of *osk* mRNP Particles Requires Microtubules

Injection of the microtubule-destabilizing drug, colcemid, into the oocyte abolished almost all fast, directed movements of the particles, which instead underwent random Brownian oscillations (Movie S6). Feeding flies with colcemid caused a similar cessation of the movement of the GFP-Stau particles and abolished their accumulation at the posterior pole (Figure S2A). The fast movements of the GFP-Stau particles resumed after the colcemid was inactivated with a short UV-pulse, and the fusion protein showed a visible accumulation at the posterior pole within 50 min (Figures S2B and S2C and Movie S7). Neither feeding, immersion of the ovaries, nor direct injection of Latrunculin A into the oocyte stopped the motility of GFP-Stau and *oskMS2* particles (Movie S8). Thus, most, if not all, of the fast particle movements are microtubule rather than actin dependent.

The results above indicate that *osk* mRNA particles are transported along microtubules, strongly suggesting that these movements represent the microtubule-dependent step in its posterior localization. However, the behavior of the particles does not correspond to the predictions of the main models for the localization of the mRNA. The movement of the particles in all directions rules out the model in which kinesin transports *osk* mRNA along a highly polarized microtubule cytoskeleton to the posterior. Two aspects of our results also argue against the cortical exclusion model. First, *osk* mRNP particles frequently undergo fast, directed movements to the posterior pole (for example, Figure 1L), indicating that they are not simply transported away from the anterior and lateral cortex. Second, the *osk* mRNP particles show a similar frequency of fast microtubule-dependent movements close to the posterior pole as they do in other areas of the oocyte, indicating that a significant number of microtubules extend into this region. Finally, the active transport of the particles suggests that the mRNA does not localize by passive diffusion and anchoring. However, random fast movements could facilitate the diffusion of the mRNA throughout the oocyte so that it can be efficiently captured by a posterior anchor.

### *osk* mRNA Particle Movement Shows a Weak Posterior Bias

To determine whether there are any anterior-posterior biases in the behavior of *osk* mRNP particles that might contribute to their delivery to the posterior, we analyzed the particle movements in more detail. We first determined the proportion of particles undergoing active transport at any moment by tracking all moving and “nonmoving” particles in several oocytes. Most particles move along irregular paths at the same speed as the yolk granules in the surrounding cytoplasm (≤0.1 μm/s), indicating that they are being carried by the Khc-dependent slow seething of the cytoplasm. However, in any given 5 s period, 13.4% of the *oskMS2* particles (91/689 particles, 7 movies) and 13.1% of the GFP-Stau particles (178/1111, 6 movies) move in a directed and processive manner at speeds that are indicative of active transport (See Figures 1E–1L).

Because we needed to image as quickly as possible to detect the moving particles, we could only collect data in a single focal plane and could therefore follow only a segment of the trajectory of most fast-moving particles. Nevertheless, both GFP-Stau and *oskMS2* particles showed similar average track lengths (2.4 and 2.8 μm, respectively), and the longest in focus movements were over 10 μm, indicating that the transport is processive. There were no significant differences between the track lengths of particles moving toward the anterior and those moving toward the posterior (Table S1).

**Figure 2:**
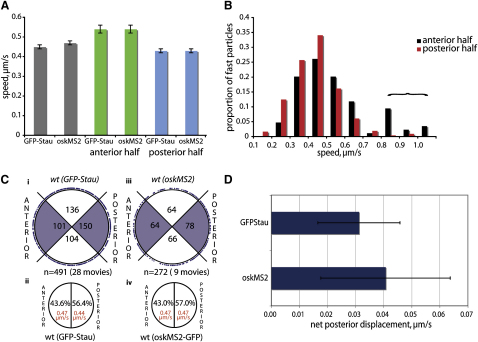
*osk* mRNA Particle Movements Show a Weak Posterior Bias (A) A graph showing the average speeds of GFP-Stau and *oskMS2*/MS2-GFP particle movements in the oocyte and in the anterior and posterior halves of the oocyte. The error bars show the standard error of the mean (SEM). (B) The distribution of GFP-Stau particle speeds in the anterior (black) and posterior (red) halves of the oocyte. (C) Circular graphs showing the orientation of GFP-Stau (i and ii) and *oskMS2*/MS2-GFP (iii and iv) particle tracks. Both methods reveal a significant posterior bias in the direction of particle movements (p < 0.005 and p < 0.025, χ^2^ test). (D) The posterior bias of particle movement results in an overall positive net posterior displacement of GFP-Stau and *oskMS2*/MS2-GFP particles in the oocyte. Error bars show SEM.

Both GFP-Stau and *oskMS2*/MS2-GFP particles also moved with very similar average velocities toward the anterior or posterior of the oocyte (Table S1). In both cases, however, particles in the anterior half of the oocyte moved with a higher average speed than those in the posterior half (Table S1). Plotting the velocity distributions of particles in both halves of the oocyte revealed that particles in the anterior have a bimodal distribution of velocities, indicating that a small population of particles move considerably faster than the rest (Figure 2B). Since *osk* mRNA is transported from the nurse cells into the oocyte by dynein, this fast population may represent particles that have just entered the oocyte and are still coupled to this pathway (Bullock and Ish-Horowicz, 2001; Clark et al., 2007).

We used the angle of the vector between the start and finish of each particle movement as a measure of their net direction and compared the number of particles traveling toward the posterior or the anterior (Figure 2C). The number of tracks in each 180° sector showed a significant excess of movements toward the posterior, with 56.5% (277/491, p < 0.005) of the GFP-Stau tracks and 57% (155/272, p < 0.025) of *oskMS2*/MS2-GFP tracks having a posterior orientation (Figure 2C, ii and iv; Table S1). We used the direction and velocity of each particle from the tracking data to calculate the net displacement for the average particle. This gives a net posterior displacement of 0.03 ± 0.01 μm/s for GFP-Stau particles and 0.04 ± 0.02 μm/s for *oskMS2*/MS2-GFP particles (Figure 2D; Table S1). The bias therefore gives the transport of *osk* RNPs an overall posterior vector.

### *mago*, *btz*, and *TmII* Mutants Reverse the Directional Bias in *osk* mRNA Movement

The results above raise the question of whether the weak posterior bias in *osk* mRNP particle movement is necessary for the posterior localization of the mRNA, or whether this active transport merely facilitates the diffusion of the mRNA so that it can be efficiently trapped at the posterior. To distinguish between these models, we examined the effects of mutants that disrupt *osk* mRNA localization. If the bias is important, one would expect it to be altered in some of these mutants, whereas the facilitated diffusion and anchoring model predicts that mutants should disrupt either the motility or the anchoring, but not the bias itself.

**Figure 3:**
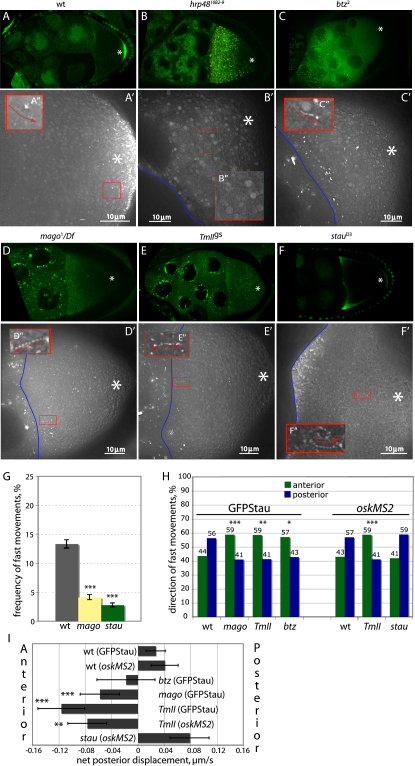
Behavior of *osk* RNP Particles in Localization Mutants (A–F″) Low magnification images (A–F) and overlays of 25 frames from high-magnification timelapse movies of *oskMS2* (A and F) or GFP-Stau (B–E). The blue lines indicate the nurse cell/oocyte boundary. The posterior pole of the oocyte is marked with white asterisks. (A–A″) Wild-type. (A″) shows a closeup of the track marked by the red rectangle in (A′). (B–B″) *hrp48*^10B2-9^ germline clones. There are no detectable particles in this mutant. (C–C″) *btz*^2^ germline clones. (D–D″) *mago*^1^*/Df(2R)F36*. (E–E″) *TmII*^gs^. (F–F″) *stau*^D3^. (G) A graph showing the proportion of particles that undergo fast, directed movements in a normalized area of cytoplasm in wild-type, *stau*, and *mago* mutant oocytes. Error bars show SEM. (H) A bar chart showing the percentages of fast particle movements toward the anterior and posterior of the oocyte in wild-type, *mago*, *btz^2^, TmII^gs^*, and *stau* mutants. The asterisks indicate the mutants in which the bias differs significantly from wild-type. (χ^2^ test: ^∗^p ≤ 0.05; ^∗∗^p ≤ 0.01; ^∗∗∗^p ≤ 0.001.) (I) A bar chart showing the net posterior displacement in wild-type, *mago*, *btz^2^, TmII^gs^*, and *stau* mutants. Error bars show SEM. (^∗^p ≤ 0.05; ^∗∗^p ≤ 0.01; ^∗∗∗^p ≤ 0.001.)

No GFP-Stau particles are visible in the oocyte cytoplasm of germline clones of *hrp48^10B2-9^* or *hrp48^7E7-18^* (Figures 3A–3B). Thus, HRP48 appears to be essential for the formation of *osk* mRNA transport particles, which may account for the uniform distribution of the mRNA in these mutants.

In contrast, particle formation appears normal in *btz*, *mago*, *TmII*^gs^ mutants. In these mutants, *osk* mRNA forms a diffuse gradient extending away from the anterior cortex of the oocyte, when visualized either by in situ hybridization or by GFP-Stau or *oskMS2* (Figures 3C–3E). There is about a 5-fold reduction in the proportion of particles that undergo fast, directed movements in these mutants, although there is still a significant amount of active transport in each case (Figure 3G, Table 1, and Movies S9, S10, and S11). Thus, the EJC and Tropomyosin affect the behavior of the *osk* RNP particles but are not required for their assembly.

To determine whether any of these mutants also affect the directional bias, we tracked a large number of particles and measured the vector of their movement. This revealed that *mago*, *btz*, and *TmII*^gs^ mutants cause a highly significant reversal in the directional bias, with 57%–59% of particles moving anteriorly, compared to only 43% in wild-type (Figures 3H and 3I). This results in a net anterior displacement of the particles that may explain the steady-state localization of the mRNA at the anterior of mutant oocytes.

### Stau Does Not Affect the Speed and Directionality of Particle Movements

*stau* mutants also contain normal numbers of *oskMS2*/MS2-GFP particles, but the majority of these are tightly localized at the oocyte anterior in a narrower region than the shallow anterior gradient seen in *mago*, *btz*, and *TmII*^gs^ mutants (Figure 3F; Movie S12). In addition, a trace amount of *osk* mRNA always localizes to the posterior of *stau* mutant oocytes at stage 9 (van Eeden et al., 2001).

As in the other localization mutants, the frequency of fast, directed *oskMS2* particle movements in the oocyte cytoplasm is reduced about 4-fold in *stau* null mutants (Figure 3G). Despite the visible anterior accumulation of *oskMS2*/MS2-GFP, there is no change in the directional bias of particle movements, with 59% moving toward the posterior, resulting in a positive net posterior displacement of the particles of 0.08 μm/s (Table 1). Most *osk* mRNP particles appear to be trapped at the anterior of the oocyte, but those that escape the anterior move with a similar average velocity (0.47 μm/s) and posterior bias to wild-type. Since Stau is required for the posterior anchoring and translation of *osk* mRNA, the weak accumulation of the mRNA at the posterior of *stau* oocytes at stage 9 cannot be explained by random transport and localized anchoring. This therefore suggests that this posterior enrichment is caused solely by the posterior bias in particle movements.

### Most *osk* RNP Particles Move toward Microtubule Plus Ends

The discovery that *osk* RNP particles move in all directions with a slight posterior bias raises the question of how this bias arises. This is difficult to address because the organization of the microtubules in the oocyte is unclear, but one can imagine two extreme scenarios. In the first, the microtubule cytoskeleton is highly polarized along the anterior-posterior axis with the plus ends at the posterior, but the particles undergo transport toward both the plus and minus ends of the microtubules, with a slight excess of plus end-directed transport. This type of bidirectional transport has been observed for several organelles, such as mitochondria in axons, and lipid droplets and pair rule transcripts in the *Drosophila* embryo (Bullock et al., 2006; Welte, 2004). At the other extreme, *osk* RNP particles could undergo exclusively plus end-directed transport along a weakly polarized microtubule cytoskeleton, in which microtubules extend in all directions, with slightly more having their plus ends pointing posteriorly.

**Figure 4:**
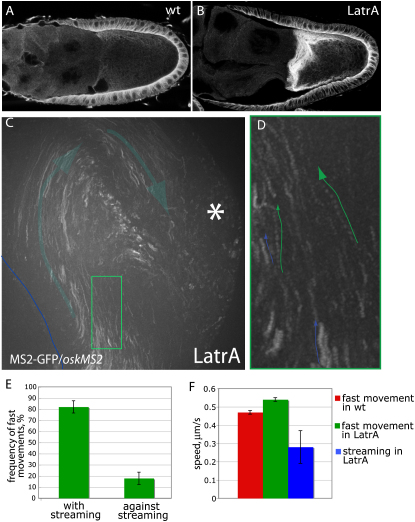
Most *osk* mRNA Particles Move toward the Microtubule Plus Ends (A and B) α-tubulin stainings to show the shallow gradient of microtubules in a wild-type oocyte (A) and the cortical microtubule bundles induced by treatment with Latrunculin A (B). (C) An overlay of 25 sequential images of a movie of a Latrunculin A-treated oocyte. All of the passively diffusing particles and most fast-moving particles move in the direction of streaming, which was followed by tracing yolk particles. (D) Two examples of fast-moving particle tracks (green arrows) from the area highlighted by the green rectangle in (C). The blue arrows show particles moving with the flow. (E) A bar chart showing the ratio of fast *oskMS2/*MS2-GFP particle movements in the same direction or the opposite direction to the cytoplasmic flows. Error bars show SEM. (F) A box plot comparing the average speed of particle movements in wild-type and Latrunculin A-treated egg chambers and the speed of streaming measured by following yolk vesicles. Actively transported particles move significantly faster then yolk vesicles. Error bars show SEM.

The models for the bias in *osk* mRNP movement can be distinguished by quantifying the proportion of particles that move toward the plus or minus ends of the microtubules. Although this is not possible in wild-type oocytes because of the complexity of the microtubule organization, treatments with actin-depolymerizing drugs induce premature streaming of the oocyte cytoplasm, which washes the microtubules into alignment around the oocyte cortex (Manseau et al., 1996). Cytoplasmic streaming requires kinesin and is thought to result from the plus end-directed transport of large organelles or vesicles (Dahlgaard et al., 2007; Serbus et al., 2005). The parallel arrays of microtubules generated by the flows are therefore aligned with their plus ends pointing in the direction of the flow. We took advantage of this to examine the movement of *osk* mRNP particles along microtubules of known polarity by imaging *oskMS2* in wild-type oocytes that had been treated with Latrunculin A (Movie S8). As shown in Figure 4E, 82% of the fast-moving particles move in the same direction as the flow. Assuming that Latrunculin does not affect the behavior of *osk* mRNP particles, this suggests that the vast majority of movements are plus end directed.

**Figure 5:**
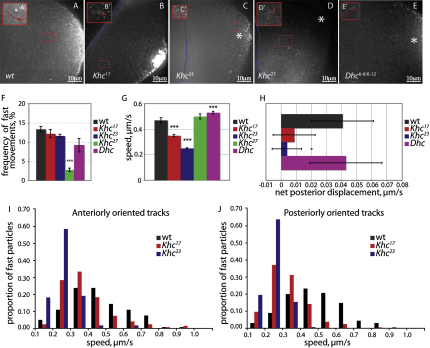
Slow *kinesin* Mutants Reduce the Speed of the Anterior and Posterior *osk* mRNA Particle Movements (A–E) An overlay of 25 sequential images to illustrate the fast-directed movements of *osk* mRNA particles in wild-type (A), in germline clones of *Khc^17^* (B), *Khc^23^* (C), and *Khc^27^* (D), and in *Dhc^6-6^*/*Dhc^6-12^* (E). Note that *oskMS2/*MS2-GFP localizes to the posterior pole in *Khc^17^* and *Khc^23^*, but this localization is slower than in wild-type. (A′)–(E′) show close-ups of the tracks highlighted by red rectangles in (A)–(E). (F) A graph showing the frequencies of fast particle movements in kinesin and dynein mutants. Error bars show SEM. (G) A graph showing the speed of *oskMS2/*MS2-GFP particle movements in kinesin and dynein mutants. Error bars show SEM. (H) A bar chart showing the average net posterior displacement in wild-type, *Khc^17^*, *Khc^23^*, and *Dhc^6-6^*/*Dhc^6-12^*. Error bars show SEM. (I and J) The distribution of velocities of particles moving toward the anterior (I) or posterior (J) in wild-type, *Khc^17^*, and *Khc^23^* mutants.

The obvious candidate for a plus end-directed motor that moves *osk* mRNPs is kinesin 1. We therefore imaged particle movement in *Khc*^27^ germline clones (Movie S13). The proportion of particles that showed fast, directed movements during a 5 s period was only 2.4%, which is more than a 5-fold reduction compared to wild-type (Figures 5A–5F). Some movements can still be detected, however, indicating that other motors can move the particles processively (Table 1).

Although these data indicate that more than 80% of *osk* mRNA particle movements are kinesin dependent, this does not necessarily mean that kinesin directly transports the particles because the loss of kinesin could affect the activity of other motors. For example, kinesin is required for the localization of cytoplasmic dynein to the posterior of the oocyte, suggesting that the two motors are associated (Januschke et al., 2002; Palacios and St Johnston, 2002). Thus, the removal of the Khc could alter dynein function, either by disrupting a kinesin/dynein complex or by disrupting dynein localization.

We therefore examined the behavior of *oskMS2* particles in *Dhc^6-6^*/*Dhc^6-12^*, a viable mutant combination that significantly delays the localization of *gurken* mRNA and halves the speed at which dynein transports mRNAs in the nurse cells (Clark et al., 2007; MacDougall et al., 2003; Mische et al., 2007). The posterior localization of *oskMS2* was not affected in this mutant combination, and the particles underwent fast movements toward the anterior and posterior of the oocyte with similar frequencies to wild-type, giving a normal net posterior displacement (Figures 5E–5H; Table 1). The speed of particle movement in both directions was slightly increased compared to wild-type (0.53 μm/s). Since one would expect the dynein hypomorphs to reduce the speed of any dynein-dependent particle movements, these data argue against a direct role for dynein in the transport of *osk* mRNPs within the oocyte.

To address the role of kinesin in *osk* mRNA transport without the complications of indirect effects caused by the null allele, we examined two missense mutations in the kinesin motor domain, *Khc^17^* and *Khc^23^*, which reduce the speed of the motor without having any detectable effects on its other functions (Brendza et al., 1999; Serbus et al., 2005). As previously reported, *osk* mRNA still localizes in germline clones of both alleles, but there is a delay in its posterior accumulation (Figures 5B and 5C). Unlike the *Khc* null mutation, *Khc^17^* and *Khc^23^* have little effect on the frequency of particle movements, consistent with idea that the mutant motor proteins associate normally with their cargoes (Figure 5F). More importantly, there is a significant reduction in the speed of *oskMS2* particle movement in these mutants, with values of 0.35 μm/s (p < 0.001) for *Khc*^17^ and 0.25 μm/s (p < 0.001) for *Khc*^23^ (Table 1; Figure 5G). This slower movement proves that kinesin transports *osk* mRNP particles along microtubules.

Since the reduced speed in *Khc^17^* and *Khc^23^* provides a direct indication of kinesin-dependent transport, it allowed us to ask whether kinesin moves *osk* mRNP particles specifically toward the posterior of the oocyte, or both anteriorly and posteriorly. The velocity profile of particles moving toward the anterior showed a pronounced shift to lower speeds in both mutants that was indistinguishable from that seen in particles moving toward the posterior (Figures 5I and 5J). Thus, kinesin mediates the majority of fast particle movements in both directions, indicating that the posterior bias is chiefly due to a weak bias in the orientation of the microtubules, rather than bidirectional transport along a more strongly polarized cytoskeleton.

## Discussion

The mechanism of *osk* mRNA localization has been controversial, and a number of competing models have been proposed to explain its targeting to the posterior. Here, we have observed directly how the RNA travels to the posterior by tracking the movements of *osk* mRNA particles at high temporal resolution in living oocytes. Surprisingly, our results are incompatible with the existing models, leading us to propose a new mechanism for the localization of the mRNA.

First, it is clear that the mRNA is not transported in a highly directed fashion toward the posterior since the particles move in all directions with only a slight posterior bias. Second, the rapid transport of the *osk* mRNPs argues against a role for passive diffusion (Glotzer et al., 1997). Third, our results are inconsistent with the two-step model for *osk* mRNA localization, in which kinesin first transports the RNA away from the anterior and lateral cortex to the oocyte center before it is translocated to the posterior in a second step (Cha et al., 2002). *osk* mRNP particles show a similar behavior in all regions of the oocyte at stage 9, with a consistent small excess of particles moving posteriorly, and this is incompatible with the idea that particles are first transported to the center. Moreover, slow *kinesin* mutants have an identical effect on the speeds of particle movements in all regions of the oocyte, strongly arguing that kinesin transports the mRNA in a one-step pathway all of the way to the posterior pole.

Instead, our data suggest that *osk* mRNA is localized by a biased random walk, in which each particle undergoes a large number of active movements in many different directions, with a small excess of movements toward the posterior. After hundreds of movements, the 14% excess of posterior movements results in a large net posterior displacement that delivers the mRNA to its destination. Given that 13% of particles are moving at any one time, the average *osk* mRNP will undergo a net posterior displacement during the 6–10 hr of stage 9 of 112–187 μm (6–10 × 3600 s × 0.13 × 0.04 μm/s). Since this is more than 1.5 times the length of the oocyte (80 μm), this is more than sufficient to produce a robust posterior localization of *osk* mRNA by the end of stage 9.

This model is supported by the observation that the direction of the bias correlates perfectly with the site of *osk* mRNA accumulation: wild-type oocytes show a posterior bias and posterior localization of the mRNA, whereas the bias is reversed in *mago*, *TmII*, and *btz* mutants, and *osk* mRNA accumulates at the anterior. The effectiveness of the biased random walk in localizing the RNA is even more clearly demonstrated by *stau* mutants: most of the mRNA is trapped at the anterior of *stau* mutant oocytes, and the mRNA that is released into the oocyte cytoplasm moves four times less frequently than in wild-type. Nevertheless, the small number of movements that occur show a normal posterior bias, which leads to a transient posterior enrichment of the mRNA that is lost at later stages because the RNA is not anchored.

Similar biased bidirectional transport has been described for lipid droplets in the *Drosophila* embryo and for many other particles and organelles in other systems (Welte, 2004). In most cases, the bias depends on the competing activities of motors that move in opposite directions. By contrast, our results indicate that the vast majority of *osk* mRNA movements are directed toward microtubule plus ends and are mediated by kinesin. First, when the microtubules are aligned around the cortex by premature cytoplasmic streaming, over 80% of fast-moving particles move in the same direction as the cytoplasmic flows, i.e., toward the plus ends. Second, more than 80% of movements are abolished by null mutations in the Khc. Third, point mutations in kinesin reduce the speed of particle movements in all directions. Fourth, we have never observed any particles that show a clear 180° reversal in the direction of their movement out of more than 3000 particle tracks analyzed, indicating that particles rarely switch between plus and minus end-directed motion.

The traditional view of the oocyte microtubule cytoskeleton is that it is polarized along the anterior-posterior axis with minus ends at the anterior and plus ends at the posterior. This view is based on the localization of fusion proteins containing the motor domains of Nod and kinesin to the anterior and posterior of the oocyte, respectively, and the assumption that these act as minus and plus end markers (Clark et al., 1994, 1997). The microtubule organization appears much more complex, however, when visualized directly: the microtubules appear to be nucleated from both the anterior and lateral cortex and extend in all directions to form an anterior-posterior gradient (Cha et al., 2001; MacDougall et al., 2003). Our data are consistent with this latter view because *osk* mRNA particles move in all directions in every region of the oocyte. More importantly, because almost all *osk* mRNA movements are plus end directed, each RNA track provides a snapshot of the polarity of a microtubule segment. The observation that 57% of tracks have a net posterior vector therefore indicates that the microtubules have only a weak orientation bias toward the posterior. Even if all 10%–20% of kinesin-independent *osk* mRNA movements are minus end directed, this would still give a posterior bias in microtubule polarity of only 62%. Thus, our data suggest a revised view of the organization of the cytoskeleton, in which the microtubules extend in all directions from the anterior and lateral cortex, with about a 20% excess of microtubules with their plus ends pointing posteriorly. One appealing aspect of this model is that it can reconcile the two opposing views of the microtubule organization. Kinβgal is an unregulated motor that constitutively moves toward the plus ends of microtubules, and we propose that it accumulates at the posterior by following a biased random walk similar to *osk* mRNA. According to this view, it is not a marker for microtubule plus ends but for regions where plus ends are most enriched.

Mutants in different components of the *osk* mRNA localization complex produce very similar phenotypes when analyzed by in situ hybridizations to fixed samples. However, they have different effects on the dynamics of *osk* mRNA particles. First, *hrp48* mutants abolish the formation of visible *osk* mRNA particles, indicating a requirement for this HnRNPA/B-like protein in the assembly of functional transport particles.

Mutants in the EJC components, Mago nashi and Btz, do not affect *osk* mRNP particle formation but reduce the frequency of particle movement and reverse the bias, so that the particles accumulate at the oocyte anterior. This behavior is what one would expect if the movement is primarily mediated by a minus end-directed motor. Furthermore, the anterior accumulation of *osk* mRNA in these mutants resembles that of many other mRNAs that are transported into the oocyte by the dynein/Bic-D/Egl pathway, and which are thought to localize to the anterior by default, because this pathway remains active in the oocyte (Serano and Cohen, 1995). Thus, the EJC may be required to turn off the dynein/Bic-D/Egl pathway when *osk* mRNA enters the oocyte so that it can then associate with the kinesin pathway.

*TmII* mutants have the same effects on *osk* mRNP dynamics as EJC mutants, suggesting that Tropomyosin is required for the same step in localization. This raises the possibility that Tropomyosin plays a role in either the recruitment of the EJC to *osk* mRNA or the subsequent activity of the EJC in switching from the anterior to the posterior localization pathway.

Stau seems to function downstream of the EJC since *osk* mRNA is either trapped at the anterior or moves with a normal posterior bias toward the posterior pole. Our results suggest that Stau regulates several aspects of *osk* mRNA behavior once it enters the oocyte. First, it seems to be required for the efficient release of the mRNA from the anterior. This may reflect a role of Staufen in the coupling of the mRNA to the kinesin-dependent posterior transport pathway. *osk* mRNA particles that escape the anterior move with a normal bias but a reduced frequency, suggesting that Stau is also required for full kinesin activity. Finally, Stau is essential for the activation of *osk* mRNA translation once the mRNA has reached the posterior pole (Micklem et al., 2000). Thus, the in vivo analysis of *osk* mRNA dynamics reveals that different components of the *osk* mRNP complex are required for at least three distinct steps in the localization pathway, namely particle formation, uncoupling from the dynein/BicD pathway, and release from the anterior and coupling to the kinesin pathway, and this may begin to explain why so many *trans*-acting factors are required for the localization of this mRNA.

The dynamics of *osk* mRNA particles have several features in common with the behavior of MS2-labeled mRNAs in mammalian cells. Fusco et al. (2003) found that RNA particles undergo stochastic movements in COS cells, in which they switch between fast microtubule-dependent movements, diffusion, and stationary phases. Furthermore, an RNA containing the *β-actin* localization signal showed a 5-fold higher frequency of fast movements than a random RNA. This is very similar to the behavior of *osk* mRNA, which undergoes fast, direct movements 4–5 times more frequently in wild-type oocytes than in *EJC, TmII*, and *stau* mutants. MS2-GFP has also been used to image CamKIIα mRNA in the dendrites of cultured neurons and labels particles that show similar bidirectional movements to *osk* mRNA (Rook et al., 2000; Kanai et al., 2004). Since dendrites contain microtubules of mixed orientations and Stau, Barentsz, and kinesin have been implicated in dendritic mRNA localization, it will be interesting to determine whether *CamKIIα* mRNA localizes by a biased random walk similar to *osk* mRNA (Kanai et al., 2004; Macchi et al., 2003).

## Experimental Procedures

### *Drosophila* Stocks

GFP-Stau was expressed from the maternal α4-tubulin promotor from a transgene on an X chromosome that also carries hsFLP (Martin et al., 2003).

To generate the *oskMS2* construct, we inserted 10 MS2-binding sites into an Spe1 site that was introduced immediately after the *osk* stop codon in a full-length *osk* genomic rescue construct (Munro et al., 2006). *oskMS2* transgene inserts were recombined with MS2-GFP transgenes on the same chromosome (Forrest and Gavis, 2003).

Germline clones were generated using the ovoD/Flp system by heat-shocking second to third instar larvae for 2 hr at 37°C for 3 consecutive days (Chou and Perrimon, 1996).

Other fly strains used were *FRT82B btz^2^* (Palacios and St Johnston, 2002), *TmII^gs^* (Erdelyi et al., 1995), *mago^1^/Df(2R)F36* (Boswell et al., 1991), *FRT 42B c Khc^27^/CyO, FRT 42B c Khc^17^/CyO, FRT 42B c Khc^23^/Cyo* (Brendza et al., 2000), *FRT40A hrp48^10B2-9^/CyO*, *FRT40A hrp48^7E7-18^/CyO* (Huynh et al., 2004), *osk^A87^* /*Df(3R)pXT103* (Jenny et al., 2006), and *Dhc*^6-6^/*Dhc*^6-12^ (McGrail and Hays, 1997).

### Fast Imaging and Deconvolution

Egg chambers were dissected directly onto coverslips in 10S Voltalef oil (Altachem). Imaging was performed on a wide field DeltaVision microscope (Applied Precision). Out-of-focus light was reassigned to its point of origin by iterative deconvolution.

### Particle Tracking and Image Analysis

Moving particles were tracked manually using the Metamorph (Universal Imaging Corporation) image analysis software. To avoid bias in the selection of particles, we tracked all visible particles in each movie. We analyzed an average of 240 particle tracks per genotype from 3 to 30 oocytes. A custom program was used to calculate the average speed, distance, and directionality of each track from the original tracking data. A vector was taken between the first and last points of each track to calculate the overall direction of the movement. The proportion of moving particles was calculated by counting the number of particles in a selected 300 × 300 pixel region (19.89 μm × 19.89 μm) and determining how many of these particles underwent active movements during the next 10 frames.

The comparisons of speeds and net posterior displacements were performed using t tests and Wilcoxon rank-sum tests. The normality of the distribution was tested using the Jacque-Bera normality test. When normal, the significance of the difference in the means was assessed using a standard Student's t test for distributions with equal variance, or the Welch-Satterthwaite-corrected Student's t test for distributions with unequal variance. When the distribution was non-normal, we compared medians using the nonparametric counterpart of the Student's t test, the Wilcoxon rank-sum test.

The difference in the posterior versus anterior bias was assessed using the standard χ^2^ test. To test whether the net posterior displacement in wild-type was significantly larger than zero we performed a Wilcoxon 1-sample test. All tests were performed using the Matlab, Minitab, and Excel software.

### Drug Treatments

The microtubule cytoskeleton was depolymerized by treating egg chambers with colcemid or colchicine (Sigma). Wild-type flies were starved for 1 day after hatching and then fed fresh yeast paste containing 100 μg/ml colcemid overnight (Pokrywka and Stephenson, 1995). Colcemid was inactivated by a 10 s pulse of UV light (Theurkauf and Hazelrigg, 1998). For immersion experiments, we dissected ovaries in 20 μg/ml colcemid in Schneider's media. Colchicine was injected into the oocyte at 100 μg/ml in water.

F-actin was depolymerized either by feeding females 200 μg/ml Latrunculin A in yeast paste or by dissecting ovaries in 50 μg/ml Latrunculin A.

## Figures and Tables

**Table 1:** GFP-Stau and *oskMS2*/MS2-GFP Particle Behavior in Localization Mutants

Phenotype	Number of Tracks	Number of Movies	Track Distance, μm	Average Speed, μm/s	Percent Tracks to Anterior	Percent Tracks to Posterior	Net Posterior Displacement, μm/s
WT (GFP-Stau)	491	28	2.43 (0.07)	0.45 (0.01)	43.6	56.4	+0.03 (0.01)
*btz*^2^ (GFP-Stau)	77	20	2.88 (0.19)	0.53 (0.03)	57.1	42.9	−0.02 (0.04)
*TmII*^gs^ (GFP-Stau)	150	23	1.83 (0.09)	0.77 (0.03)^∗∗∗^	58.7	41.3	−0.12 (0.04)^∗∗∗^
*mago* (GFP-Stau)	192	30	1.84 (0.1)	0.62 (0.02)^∗∗∗^	58.9	41.1	−0.057 (0.03)^∗∗∗^

WT (*oskMS2*/MS2-GFP)	272	9	2.85 (0.11)	0.47 (0.01)	43.0	57.0	+0.04 (0.02)
*TmII*^gs^(*oskMS2*/MS2-GFP)	199	17	2.09 (0.08)	0.55 (0.02)^∗∗^	58.8	41.2	−0.08 (0.03)^∗∗^
*stau* (*oskMS2*/MS2-GFP)	152	31	3.00 (0.1)	0.47 (0.01)	40.8	59.2	+0.08 (0.03)
*Khc*^27^ (*oskMS2*/MS2-GFP)	154	19	2.37 (0.09)	0.50 (0.02)	49.4	50.6	+0.02 (0.03)
*Khc*^17^ (*oskMS2*/MS2-GFP)	272	8	2.59 (0.09)	0.35 (0.01) ^∗∗∗^	43.8	56.2	+0.01 (0.01)
*Khc*^23^ (*oskMS2*/MS2-GFP)	231	8	3.01 (0.11)	0.25 (0.004)^∗∗∗^	47.2	52.8	+0.004 (0.01)^∗^
*Dhc*^6-6^/^6-12^ (*oskMS2*/MS2-GFP)	231	21	3.06 (0.11)	0.53 (0.01)^∗∗∗^	42.7	57.3	+0.04 (0.02)

Values shown are means for all particles that move faster than cytoplasmic flows (>0.1 μm/s) with the SEM in the parentheses. Statistically significant differences between the behavior of particles in wild-type and a given mutant are shown by asterisks: ^∗^p ≤ 0.05; ^∗∗^p ≤ 0.01; ^∗∗∗^p ≤ 0.001. Corresponding p values are shown in parentheses. WT: wild-type.

## Supplemental Data

Document S1. One Table and Two Figures

Movie S1. Timelapse Movie of GFP-Stau Particles in a Wild-Type Chamber The movie shows the movements of GFP-Stau particles and their localization to the posterior pole. The small bright particles are GFP-Stau, whereas the large grey round structures are yolk vesicles that can be also seen by wide-field imaging. In this and all subsequent movies, the posterior pole of the oocyte is to the right. The movie consists of 60 sequential time points, which are 0.478 s apart played at 20 fps. Magnification: 2000×. The second part of the movie shows a closeup of the region of cytoplasm highlighted by the black rectangle. The tracks of some of the particles are shown on a repeat of this movie.

Movie S2. GFP-Stau Particles Moving along the Same Track A magnified area of the oocyte cytoplasm showing two GFP-Stau particles following a similar trajectory. The movie consists of 40 sequential time points, which are 0.477 s apart. Magnification: 4000×.

Movie S3. Timelapse Movie Showing *oskMS2*/MS2-GFP Particle Movements in a Wild-Type Oocyte The movie consists of 120 sequential time points, which are 0.672 s apart played at 20 fps. Magnification: 2000×.

Movie S4. *oskMS2*/MS2-GFP Particles Moving along the Same Track A magnified area of the oocyte cytoplasm to show two *oskMS2*/MS2-GFP particles following a similar trajectory. Some diffusive particles undergoing Brownian movements are also highlighted by colored tracks.

Movie S5. *oskMS2*/MS2-GFP and RFP-Stau Label the Same Moving Particles Two-color timelapse movie to show the colocalization of *oskMS2*/MS2-GFP and RFP-Stau to the same moving particle. *oskMS2*/MS2-GFP is in green, RFP-Stau is in red. Due to the sequential imaging of the red and green channels, the two signals do not colocalize completely because of the movement of the particle between images. Twenty-two time frames for each channel, 1.052 s apart. Magnification: 4000×.

Movie S6. Colchicine Injection Disrupts Processive Directed Movements of GFP-Stau Particles Timelapse movies showing *oskMS2*/MS2-GFP particle movement in a wild-type oocyte prior to colchicine injection (left) and the same oocyte visualized 15 min after the injection of colchicine (right). Most particles stop moving processively and instead undergo Brownian oscillations. The left movie consists of 93 time frames played at 20 fps. Magnification: 2600×. The right movie consists of 107 time frames played at 20 fps. Magnification: 2600×.

Movie S7. Reactivation of GFP-Stau Particle Movement and Its Posterior Localization upon UV Inactivation of Colcemid A movie showing GFP-Stau particles in ovaries of females that had been fed with colcemid overnight and 50 min after the inactivation of colcemid with a 10 s UV pulse. GFP-Stau particles undergo fast processive movements after colcemid inactivation and start to accumulate at the posterior pole. Each part of the movie consists of 45 time frames, 0.999 s apart. Magnification: 2000×.

Movie S8. A Timelapse Movie of *oskMS2*/MS2-GFP Particles in a Latrunculin A-Treated Oocyte The right panel shows a temporal merge of successive frames from the movie to reveal *oskMS2*/MS2-GFP particle tracks. The second half of the movie shows an area of the cytoplasm to show particles that are moving faster than streaming and in the same direction. The movie consists of 120 frames, 0.718 s apart. Magnification 2000×.

Movie S9. Movement of GFP-Stau Particles in a *btz2* Germline Clone Timelapse movie of GFP-Stau particle movements in a wild-type oocyte (left) and a *btz^2^* mutant oocyte (right). Both movies were recorded at 0.478 s per frame.

Movie S10. Movement of GFP-Stau Particles in *mago1*/Df(2R)F36

Movie S11. Movements of GFP-Stau Particles in a *tm2gs* Germline Clone

Movie S12. Movements of *oskMS2*RNA-MS2GFP Particles in *stauD3* Mutant

Movie S13. *oskMS2*-GFP Particle Movements in a *Khc27* Germline Clone A movie showing wildtype and *Khc^27^* oocytes side by side played at the same speed (0.6s per frame). *oskMS2RNA*-MS2GFP does not localise to the posterior in *Khc^27^*, and there are few fast particle movements.
