# Supplementary material for: In Vivo Imaging of oskar mRNA Transport Reveals the Mechanism of Posterior Localization
Source: Cell. 2008 Sep 5;134(5):843–53. doi: 10.1016/j.cell.2008.06.053 (PMC2585615; doi:10.1016/j.cell.2008.06.053)
Supplement: Document S1. One Table and Two Figures [file mmc1.pdf]

Cell, Volume *134*

Supplemental Data

In Vivo Imaging of *oskar* mRNA Transport Reveals the Mechanism of Posterior Localization

Vitaly L. Zimyanin, Katsiaryna Belaya, Jacques Pecreaux, Michael J. Gilchrist, Alejandra Clark, Ilan Davis, and Daniel St Johnston

Table S1. Comparison of the dynamics of GFP-Stau and oskMS2-GFP particle movement

Phenotype	Number of tracks	Number of movies	Track distance, µm	Average speed, µm/s	Percent tracks to anterior	Percent tracks to posterior	net posterior displacement, µm/s
wt (GFP:Stau)	491	28	2.43 (0.07)	0.45 (0.01)	43.6	56.4	0.03 (0.01) p<0.01[a]
wt (oskMS2GFP)	272	9	2.85 (0.11)	0.47 (0.01) n.s	43.0	57.0	0.04 (0.02) n.s, p<0.05[a]
*osk*A87/Df (oskMS2GFP)	224	4	2.34 (0.11)	0.60 (0.01)*** p<0.001	42.0	58.0	0.04 (0.03) n.s.
wt (GFP:Stau) Anterior oriented	214		2.58 (0.12)	0.47 (0.01)			
wt (GFP:Stau) Posterior oriented	277		2.32 (0.08)	0.44 (0.01)* p=0.02			
wt (oskMS2GFP) Anterior oriented	122		2.85 (0.18)	0.47 (0.02)			
wt (oskMS2GFP) Posterior oriented	150		2.85 (0.12)	0.47 (0.01) n.s.			
wt (GFP:Stau) anterior half of the oocyte	84	22	2.80 (0.02)	0.54 (0.02)	44.1	55.9	0.05 (0.04)
wt (GFP:Stau) posterior half of the oocyte	407	24	2.35 (0.07)	0.43(0.01)*** p<0.001	43.5	56.5	0.03 (0.02) n.s.
wt (oskMS2GFP) anterior half of the oocyte	108	7	2.93 (0.19)	0.54 (0.02)	47.3	52.7	0.001 (0.03)
wt (oskMS2GFP) posterior half of the oocyte	164	2	2.80 (0.12)	0.43 (0.01)*** p<0.001	49.6	60.4	0.06 (0.02) n.s

Values shown are means for all particles that move faster than cytoplasmic flows (> 0.1 μm/s) with the SEM in the parentheses. The significance scores indicate whether the values for a particular experiment are significantly different from those of the top experiment in each segment of the Table defined by heavy black lines. Statistically significant differences between populations are shown with asterisks: $^{*}p \le 0.05$; $^{**}p \le 0.01$; $^{***}p \le 0.001$. Corresponding p values are shown in the parenthesis. [a] probability that the net posterior displacement is larger than 0.

Figure S1. *oskMS2* RNA localizes in a *osk* mRNA null mutant background.

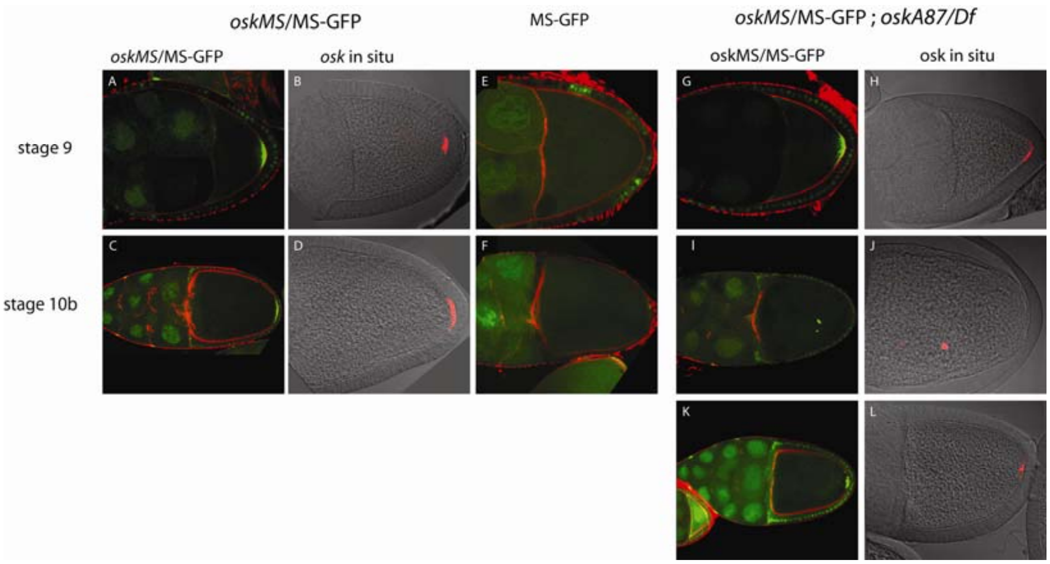

(A-D) *oskMS2/MS2-GFP* localizes to the posterior of the oocyte in wild-type. (A and C) GFP fluorescence; (B and D) *in situ* hybridization to *osk* mRNA. (E and F) MS2-GFP localizes to the nuclei in the absence of *oskMS2* mRNA.

(G-L) *oskMS2* and *MS2-GFP* were crossed into an *osk* mRNA null mutant background ($osk^{A87}/Df(3R)p^{XT103}$). (G,H) *oskMS2* RNA rescues the early oogenesis arrest of the *osk* RNA null mutant, and localizes normally to the posterior pole of the oocyte during stages 9-10. (I-L). *oskMS2* RNA falls off the posterior pole in 80% of $osk^{A87}/Df(3R)p^{XT103}$oocytes after stage 10b, indicating that it is not anchored properly in the absence of the endogenous mRNA. (G,I, and K) GFP fluorescence; (H,J, and L) *in situ* hybridization to *osk* mRNA. In (A,C,E,F,G,I, and K), actin is stained with Rhodamine-Phalloidin.

Figure S2

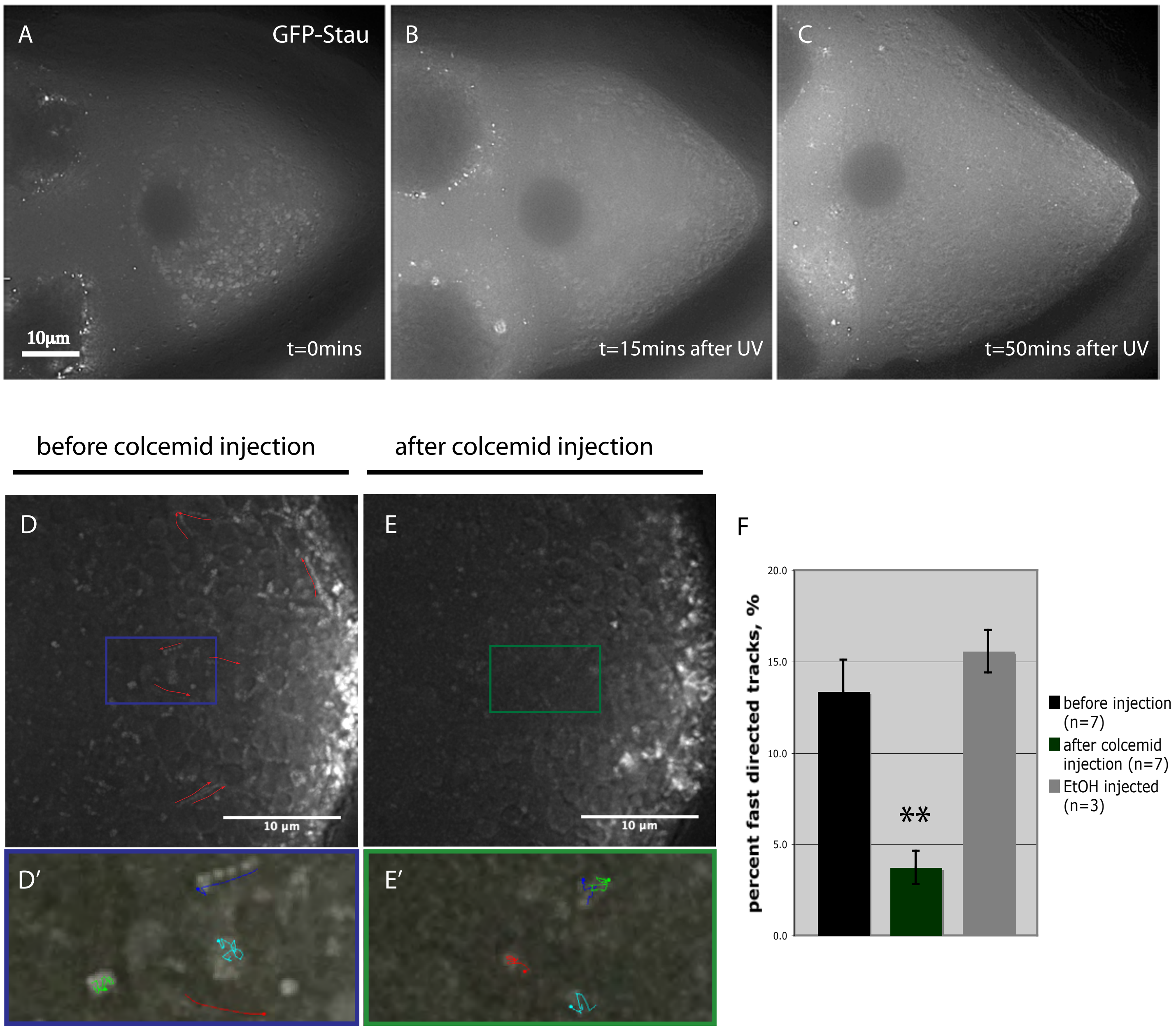
A
GFP-Stau
10µm
t=0mins
B
t=15mins after UV
C
t=50mins after UV
before colcemid injection
after colcemid injection
D
10 µm
E
10 µm
D′
E′
F
percent fast directed tracks, %
20.0
15.0
10.0
5.0
0.0
**
before injection (n=7)
after colcemid injection (n=7)
EtOH injected (n=3)

Figure S2 The movement of *osk* mRNA particles is microtubule-dependent

(A) Depolymerization of the microtubules with colcemid prevents the posterior accumulation of GFP-Stau and abolishes almost all fast particle movements in the oocyte.
(B and C) Upon inactivation of the colcemid with a short UV-pulse, GFP-Stau particles resume their movements, with visible accumulation of GFP-Stau at the oocyte posterior after 50mins.
(D and E) Injection of the microtubule-depolymerizing drug, colchicine, abolishes almost all fast particle movements. (D) An overlay of 60 frames to show fast *osk-MS2*/MS2-GFP particle movements in the oocyte before injection. The red arrows indicate fast tracks. (D') A close up of the blue box in (D) showing two fast (dark blue and red) and two diffusive tracks (light blue and green). (E) The number of fast particle movements is significantly reduced 15 min after colchicine injection, and none can be seen in the overlay of 25 frames (E) or in the close up of the area highlighted by the green rectangle (E').
(F) Quantification of the number of fast particle movements in the oocyte before and after colchicine injection.
